# Supplementary material for: Diagnostic performance of optical coherence tomography macular ganglion cell inner plexiform layer and retinal nerve fiber layer thickness in glaucoma suspect and early glaucoma patients at St. Paul’s hospital millennium medical college, Addis Ababa, Ethiopia
Source: PLoS One. 2023 Jan 31;18(1):e0263959. doi: 10.1371/journal.pone.0263959 (PMC9888678; doi:10.1371/journal.pone.0263959)
Supplement: Attachment — Submitted filename: Response to Reviewers.pdf [file pone.0263959.s002.pdf]

01-12-2021

Rebuttal letter

Dear Akram Belghith (academic editor) and dear reviewer(s)

First I would really like to thank you for your time to review the manuscript and giving detailed and supportive comments.

I've made changes and label the changes with a red color & send it as a file named 'revised research manuscript'.

I've tried to address all the major and minor comments. I've added plots of AUROC and representative OCT (optical coherence tomography) image to show how each quadrant was extracted.

Regarding calculation of sensitivity and specificity, we take examination of optic nerve head by 90diopter volk lens as **gold standard** way and we cross tabulate it with color coded result of OCT. Any of one of the quadrants with red color of either GCIPL or RNFL will be labeled as glaucomatous.

As your comment, the inner retinal thickness tends to decrease in older ages. But in our setup it was very difficult to find older people who can fulfill all of the inclusion criteria for the healthy controls so we have mentioned the unmatched age between normal control & glaucoma patients as the limitation of this research even though all the comparisons were age adjusted.
